# The Possible Role of Interleukin (IL)-18 and Nitrous Oxide and Their Relation to Oxidative Stress in the Development and Progression of Breast Cancer

**DOI:** 10.31557/APJCP.2019.20.9.2659

**Published:** 2019

**Authors:** Mona Mohamed K El-Deeb, Heba Gaber El-Sheredy, Ayman Farouk Mohammed

**Affiliations:** 1 *Department of Chemical Pathology,*; 2 *Department of Cancer Management and Research,*; 3 *Department of Surgery, Medical Research Institute, Alexandria University, Alexandria, Egypt. *

**Keywords:** Interleukin, 18- nitric oxide, cancer breast, oxidative stress

## Abstract

**Background:**

Cancer breast is the most common malignant tumor in females globally. Mechanisms linking inflammatory cytokines and tumour growth and progression have not been established. Interleukin (IL)-18 has a modifying role in the immune defense against tumor cells. It induces production of IFN-γ. It also increases the immune cells cytotoxic activity and enhances the production of other proinflammatory cytokine. Nitric oxide (NO) has both promoting and inhibiting effects on tumorigenesis. Oxidative stress is a phenomenon that leads to oxidative damage of biomolecules, mutagenesis and carcinogenesis.

**Objective:**

The purpose of this research is to identify the potential role of IL18 and NO and their relation to oxidative stress in the development of cancer breast.

**Patients and Methods:**

This study included 120 women split into two groups ; control group and patient groups that divided into: group B (30 patients with benign breast tumors), group N (30newly diagnosed cancer breast patients) ; and group M (30 metastatic cancer breast patients).

**Results:**

Serum total anti-oxidant capacity was significant high in both cancer breast groups. Total oxidative capacity was significantly higher level in metastatic group. NO levels were significantly higher values in the three cancer breast patients groups compared to control group.IL18 was significantly high in the metastatic group.

## Conclusions:

Serum IL-18 and NO activity can be used as a marker for evaluating disease activity in patients with cancer breast.

## Introduction

Cancer breast is the most common malignant tumor in females globally, with newly diagnosed cases 2,088,849 and number of deaths of 626,679 reported by GOBOCAN (2018) and Bray et al., (2018) about half of the world cancer breast cases and the majority of the cancer breast deaths were reported to occur in the developing countries (Torre et al., 2017). In Egypt cancer breast represents 38.8% of newly diagnosed cancer cases among Egyptian females (Ibrahim et al., 2014).

Breast cancer etiology and pathogenesis involve genetic, hormonal and other modifying factors. (Sotos et al., 2018) Mechanisms linking inflammatory cytokines and tumour growth and progression have not been established yet as some cytokines (IL2, IL11 and TGF beta) stimulate cancer breast growth and invasion, while others (IL12,IL 18and interferons) inhibit it (Capone et al., 2016). 

IL-18 has been found to play a dual role in cancer, as it can promote tumor development, progression, migration, invasion, and metastasis. However, it enhances anti-tumor immunity and reduces tumor growth. (Fabbi et al., 2015; Kim et al., 2009). IL-18 in combination with IL-12 can activate cytotoxic T cells (CTLs), as well as natural killer (NK) cells, to produce IFN-γ and, therefore, may contribute to tumor immunity (Esmailbeig et al., 2017; Kuppala et al., 2012). It was found that Interleukin (IL)-18 has a modifying role in the immune defense against tumor cells where it induces IFN-γ production. Moreover, it augments the cytotoxicity of immune cells (natural killer (NK) and T cells) and increases the production of other proinflammatory cytokines such as tumor necrosis factor-alpha (TNF-α ), IL-1β , IL-8, and nitric oxide (NO) (Günel et al., 2002; Eissa et al., 2005) 

However, the suppressive or promotive effects of IL-18 on carcinogenesis are not yet fully understood. Several cancers including bladder cancer (Jaiswal et al., 2013) and ovarian cancer (Samsami et al., 2009) and gastrointestinal cancer (Haghshenas et al., 2009) showed higher levels of IL-18.IL 18 and its receptor (IL18 R) expression in were found to have a role in ovarian epithelial cells either cancerous or normal (Medina et al., 2014). It has also been discovered to be a predictor of HCC patients ‘ poor outcome as IL18 R was also expressed in HCC cells (Asakawa et al., 2009, Curran et al., 2017). However IL-18 production is decreased in colon adenocarcinomas and mutations in the IL-18 receptor accessory protein (IL18RAP) gene are associated with Crohn’s disease and inflammatory bowel diseases (Mager et al., 2016) 

NO have both promoting and inhibiting effects on tumorigenesis. It causes damage to DNA and encourages angiogenesis (Choudhari et al., 2013) In contrast, by causing apoptosis, it suppresses tumor development and metastasis. The equilibrium between the two opposing roles may rely on the tumor tissue concentration of NO and its relationships with other molecules including IL-18, IFN, and TNF-α. High NO concentrations exert anti-proliferative impacts while tumor development is facilitated by low concentrations (Landskron et al., 2014; Yeon et al., 2011). 

Oxidative stress is a phenomenon in which the antioxidant mechanisms are over whelmed by excessive reactive oxygen and nitrogen species resulting in oxidative damage of biomolecules mainly lipid peroxidation, mutagenesis and carcinogenesis in particular (Yeon et al., 2011). Several studies have shown that in cancer breast patients, levels of oxidative stress markers are increased (Muraoka et al., 2003; Aghvami et al., 2006). The imbalance between oxidative stress (free radicals) and antioxidants (such as glutathione peroxidase, vit E and uric acid) in development of cancer breast has been studied on a wide base (Khanna et al., 2014). 

Chronic inflammation is the key factor responsible for promotion of cellular changes with the increase of free radicals (ROS) stimulating oxidative stress and reduce cellular antioxidant capacity (Landskron et al., 2014) Cytokine-induced oxidative stress could induce breast carcinogenesis by forming genes instability such as affecting DNA methylation that is essential in malignant transformation ( Eissa et al., 2005; Yeon et al., 2011). 

In cancer breast both estrogen and progesterone regulate NO levels.(Dias et al.,) No expression has been identified in different cancers, where high levels observed in gastric and lung cancer.(Choudhari et al.,2013) Different factors including smoking,multiparity,long-term use of oral contraceptive and chronic inflammation are suggested to increase NO levels.(Dias et al).

Although the presence of evidence suggesting the role of low-grade inflammatory marker, specifically of cytokines in the promotion, angiogenesis, and metastasis of cancer breast (Landskron et al., 2014; Yeon et al., 2011) 

Data concerning their role in relation to oxidative stress and the onset, progression and metastasis of cancer breast has not been extensively studied (Aghvami et al., 2006; Roque et al., 2015). Thus, the purpose of this study is to determine the possible role of IL18 and NO and their relation to total oxidative capacity and antioxidants in the development and progression of cancer breast in a group of Egyptian females. 

## Materials and Methods

A case-control study was conducted out on 120 females which were divided into two main groups; control group which consisting of 40 apparently healthy female volunteers of comparable age and socio-economic status with patient groups; their mean age was 45.30 ± 10.4 years.

The patient groups comprising breast tumours patients and divided into 3 groups: group B comprising 30 patients with benign non-proliferative breast tumours, group N comprising 30 newly diagnosed cancer breast patients and group M that included 30 patients with metastatic cancer breast; their mean age was (43.47 ± 9.91, 51.40 ± 10.17 and 48.60 ± 9.65 years) respectively.

From those admitted to the hospital of Medical Research Institute, Alexandria University, Alexandria, Egypt. the patient groups were chosen. Patients ‘ clinical and pathological information were gathered prospectively. According to the Helsinki declaration, a written consent was obtained from all patients and control subjects and endorsed by the Medical Research Institute’s ethical commission. Patients with any concomitant disorder that could influence the study outcomes, such as smoking, liver dysfunction, allergic, autoimmune diseases, or other systemic diseases such as diabetes mellitus were excluded. 

Benign breast tumours group included the patients who admitted to the surgical unit for breast swelling (s) and managed with excisional biopsy. The study included only the cases with non-proliferative breast tumours.

Newly diagnosed patients showed stage I-III breast cancer pathologically proven. The metastatic disease is demonstrated by either radiological studies (CT or MRI) or pathological evaluation or both. Blood samples were collected from newly diagnosed and metastatic cancer breast patients at the time of presentation before starting chemotherapy.

Clinical data of the studied groups were collected prospectively. These data included: age and menopausal status. Pathological data for the two cancerous groups(group N,M) included tumor size, nuclear grade, histological type, status and number of positive axillary lymph nodes, presence of lymphovascular invasion, status of estrogen, progesterone receptors and Her-2 (human epidermal growth factor receptor-2) expression.

After 6 hours of fasting, 7 mL of entire venous blood was removed from each female participant. The blood was left to clot, centrifuged, and the serum collected was split into 4 aliquots: the first aliquot was maintained to determine the complete serum oxidative ability (TOC), the 2^nd^ aliquot for total antioxidative capacity (TAC) measurements. TOC was measured using ELISA technique (Immundiagnostik AG, Germany) and TAC was measured using (Cell Biolabs’ OxiSelect™ Total Antioxidant Capacity (TAC) Kit . The 3^rd^ and 4th aliquots where kept for determination of nitric oxide (NO) and interleukin 18 (IL18) respectively , where Enzyme-linked immunosorbant assay (ELISA) sandwich technique was used for measurement of IL-18 (eBioscience high performance assay, Vienna, Austeria) and Griess assay method was used for determination of NO in the deproteinized serum samples 

The TAC Assay is based on the reduction of Copper (II) to Copper (I) by antioxidants such as uric acid. Upon reduction, the Copper (I) ion further reacts with a coupling chromogenic reagent that produces a color with a maximum absorbance at 490 nm. Compared with a known uric acid normal curve, the net absorbance values of antioxidants. Absorption values are proportional to the total reduction capacity of the sample. Results are expressed as “μM equivalents of copper reduction” or “mM equivalents of uric acid. Nitric oxide assay was based on reduction of nitrate to nitrite by nitrate reductase in the presence of β-nicotinamid dinucleotide phosphate (β-NADPH) where the concomitant oxidation of β- NADPH was monitored by the decrease in absorbance at 340 nm and concentrations were deduced from the appropriate standard curve and results were given as micromoles per liter .

*Statistical Analysis*

Statistical evaluation was carried out for Windows statistical software with the Statistical Package for Social Sciences (SPSS) version 20.0. With the Kolmogorov-Smirnov test, quantitative variables were tested for normality. Data revealed normal distribution was represented as mean± standard deviation, and the data showed deviation from normal distribution was represented as median and range. Variance analysis (ANOVA) or F-test is used when the information is normally distributed for comparison between more than two means. Kruskal-Wallis has been used to test the equality between groups of population medians. Abnormally quantitative data was compared using Mann Whitney test. P value of less than 0.05 was considered statistically significant. Qualitative data was expressed using number and percent and was compared using Chi square test. Spearman’s correlation test was used to investigate the relationship between non parametric quantitative variables.

## Results

Statistical analysis of the results showed no significant difference between the studied groups regarding age, but there was a statistically significant difference in the frequency of premenopausal status (P = 0.018) between the groups studied. Benign breast tumor group (B) and metastatic cancer breast group (M) (46% and 53.3% respectively) showed greater frequency relative to the control group (10%) and newly diagnosed cancer breast group (20%) (Table 1). Clinicopathological data of the cancer breast groups are summarized (Table 2).

**Figure 1:**
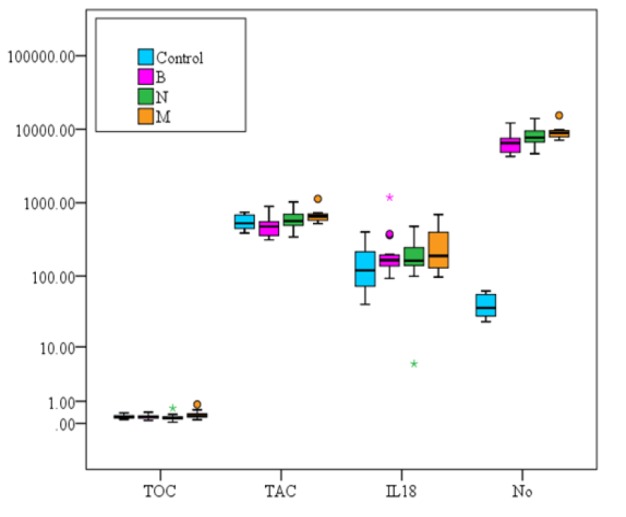
Comparison between the Studied Groups According to TOC, TAC, IL18 and No. TOC, total serum oxidative capacity; IL-18, Interleukin-8; NO, Nitric Oxide; TAC, total antioxidative capacity

Statistical analysis of the studied parameters in the four studied groups was shown in (Table 3 and Figure 1). Serum total anti-oxidant capacity (TAC) showed significantly higher level (P = 0.007) in both cancer breast groups (N and M) than in the benign group (B) while, it showed no significant difference between the three patient groups (N, M and B) and the control group. However, total oxidative capacity TOC showed only significantly higher level (P = 0.05) in metastatic group than newly diagnosed group.

The level of nitric oxide levels showed statistically significant higher values in the three cancer groups than the control group (P< 0.001) in addition the levels in cancer group(Nand M) were significantly higher than in the benign group (B) (P< 0.001). IL18 showed only significant higher levels in the metastatic patient group than the controls (P=0.05).

There was no statistical significance between any of the parameters studied when the cancer group (M + N) was classified according to HER2 status cases (Table 4).

Statistical analysis of the correlations between the different studied parameters (Tables 5, 6, 7) demonstrated positive correlation between TOC and IL18( r= 0.731, P= 0.002) and between TAC and NO (r= 1.000, P=0.04) in the benign group B, however there where negative correlation between TOC and each of TAC and NO (r=-0.648, P= 0.009 and r-0.646, P=0.008 respectively) in the newly diagnosed group N. Positive correlation between TAC and NO occurred while studying the correlation between the laboratory parameters in the whole N+M cancer group. (r=1.000, P<0.001)

## Discussion

Interleukins (ILs) are factors that stimulate many signaling pathways and regulate the transcription of certain genes involved in cellular proliferation and survival. (Gelaleti et al., 2012) They also function as immune mediators (Wilczynski et al., 2006) and were connected to cancer process by playing a main role in leukocyte recruitment and placement in carcinogenesis (Nicolini et al., 2006).

Interleukin-18 (IL-18) is an immunoregulatory cytokine that has multiple biologic functions such as promoting the production of granulocyte macrophage colony stimulating factor, IL-2 and activating NK cells and macrophages (Ling et al., 2011) The production of IL-18 may be induced in response to tumor cells or other factors related to tumor growth (Günel et al., 2003).

**Table 1:** Comparison between the Studied Groups According to Age

	Control (n = 40)	B (n = 30)	N (n = 30)	M (n = 30)	p
Age (years)	45.30 ± 10.4	43.47 ± 9.91	51.40 ± 10.17	48.60 ± 9.65	0.07

N, Newly diagnosed breast cancer; B, Benign breast condition; M, Metastatic breast

**Table 2:** Comparison between the Studied Groups According to Menstrual History

Menst. state	Control n = 40 (100%)	B n = 30 (100%)	N n = 30 (100%)	M n = 30 (100%)	p
Premenopausal	4 (10.0%)	14 (46.7%)	6 (20.0%)	16 (53.3%)	0.018*
Menopause	36 (90.0%)	16 (53.3%)	24 (80.0%)	14 (46.7%)	

**Table 3:** Clinicopathological Data in the Newly Diagnosed (N) and Metastatic (M) Breast Cancer Patients

	N n = 30 (%)	M n = 30 (100%)
Tumor size		
T0	0 (0.0)	0 (0.0)
T1	4 (13.3)	6 (20.0)
T2	22 (73.3)	20 (66.7)
T3	4 (13.3)	4 (13.3)
Lymph node		
N0	6 (20.0)	0 (0.0)
N1	10 (33.3)	10 (33.3)
N2	8 (26.7)	18 (60.0)
N3	6 (20.0)	2 (6.7)
ER.PR		
-ve	2 (6.7)	6 (20.0)
+ve	28 (93.3)	24 (80.0)
HER2		
-ve	18 (60.0)	12 (40.0)
+ve	12 (40.0)	18 (60.0)
Tumor grade		
0	0 (0.0)	0 (0.0)
1	2 (6.7)	0 (0.0)
2	22 (73.3)	22 (73.3)

N, Newly diagnosed breast cancer; B, Benign breast condition; M, Metastatic breast; ER, Estrogen receptor; PR, Progesterone receptor

**Table 4:** Comparison between the Studied Groups According to TOC, TAC, NO, IL-18

	Control (n = 40)	B (n = 30)	N (n = 30)	M (n = 30)	p
TOC	0.22 (0.13 - 0.39)	0.24 (0.10-0.42)	0.20 (0.04-0.61)	0.26^c^ (0.12-0.81)	0.05*
TAC (umol/l)	527.9 (386.6-743.5)	475.8 (312.3–892.2)	565.0^b^ (342.0–1026.0)	654.3^b^ (520.4–1130.0)	0.007*
NO	36.3 (23.2 – 62.3)	6491.4^a^ (4259.9 – 12171.9)	7708^ ab^ (4665.8 – 13977.3)	8925^ab^ (7099.9 – 15416.1)	<0.001*
IL18	119.9 (40.5 – 399.9)	164.8 (93 – 1187.2)	162.3 (5.5 – 477.2)	188.9 ^a^ (96.6 – 691.0)	0.05*

N, Newly diagnosed breast cancer; B, Benign breast condition; M, Metastatic breast; ^a^, significant with control group; ^b^, significant with B group; ^c^, significant with N group; *, Statistically significant at p ≤ 0.05; TOC, total serum oxidative capacity; IL-8, Interleukin-8; NO, Nitric Oxide; TAC, total antioxidative capacity.

**Table 5:** Relation between ER.PR and HER2 with Levels of Studied Parameters in N+M Group

	ER.PR		HER2	
	Odd ratio(95% CI)		Odd ratio(95% CI)	
	-ve	+ve	-ve	+ve
	(n = 8)	(n = 52)	(n = 30)	(n = 30)
TOC (mmol/l)	0.32 (0.22-0.42)	0.21 (0.04-0.61)	0.21 (0.04-0.81)	0.24(0.09-0.61)
P	0.127		-	
TAC (umol/l)	653.52 (342.0-1130.0)	639.40 (446.10-1026.0)	578.42 (342.0-1130.0)	654.27 (446.10-907.05)
P	0.668		0.851	
NO	8915.7 (4665.8–15416.1)	8723 (6085.9 – 13977.3)	7891.1 (4665.8 –15416.1)	8925.9 (6085.9 – 12374.5)
P	0.668		0.851	
IL18	206.2 (127.6 – 403.9)	165.3 (5.5 – 691.0)	162.6 (5.5 – 528.4)	236.1 (96.6 – 691)
P	0.95		0.485	

ER, Estrogen receptor; PR, progesterone receptor; TOC, total serum oxidative capacity; IL-8, Interleukin-8; NO, Nitric Oxide; TAC, total antioxidative capacity

**Table 6:** Correlations between Different Parameters in B Group

		TOC (mmol/l)	TAC (umol/l)	NO	IL18
TOC (mmol/l)			0.104	0.108	0.731*
	p		0.713	0.716	0.002*
TAC (umol/l)	r_s_			1.000*	0.233
	p			0.04*	0.404
NO	r_s_				0.243
	p				0.406
IL18	r_s_				
	p				

r_s_, Spearman coefficient; *, Statistically significant at p ≤ 0.05; TOC, total serum oxidative capacity; IL-18, Interleukin-8; NO, Nitric Oxide; TAC, total antioxidative capacity

**Table 7:** Correlations between Different Parameters in N Group

		TOC (mmol/l)	TAC (umol/l)	NO	IL18
TOC (mmol/l)	r_s_		-0.648*	-0.646*	0.048
	p		0.009*	0.008*	0.869
TAC (umol/l)	r_s_			1	0.164
	p			-	0.576
NO	r_s_				0.165
	p				0.572
IL18	r_s_				
	p				

r_s_, Spearman coefficient;*, Statistically significant at p ≤ 0.05;TOC, total serum oxidative capacity; IL-18, Interleukin-8; NO, Nitric Oxide; TAC, total antioxidative capacity.

**Table 8:** Correlations between Different Parameters in N + M Group

		TOC (mmol/l)	TAC (umol/l)	NO	IL18
TOC (mmol/l)	r_s_		-0.135	-0.137	0.192
	p		0.476	0.478	0.319
TAC (umol/l)	r_s_			1	0.264
	p			<0.001*	0.167
NO	r_s_				0.262
	p				0.169
IL18	r_s_				
	p				

r_s_, Spearman coefficient ; *, Statistically significant at p ≤ 0.05; TOC, total serum oxidative capacity; IL-18, Interleukin-8; NO, Nitric Oxide; TAC, total antioxidative capacity.

Nitric oxide (NO) was found to be involved in the multiple steps of tumorigenesis. High NO production levels increase vascularity of the tumor and facilitate tumor metastasis in patients with breast carcinoma (Fatehya et al., 2011).

Measurement of total tissue and plasma antioxidant capacity was commonly used in a number of human illnesses including cancer. It was suggested that the non-enzymatic antioxidant network of the body can be accessed by measuring the total ability of antioxidants (TAC). Low complete antioxidant capacity may be an indication of oxidative stress or enhanced oxidative damage susceptibility (Abdel-Salam et al., 2011). 

In the present study, our aim was to investigate the serum levels of IL-18 and NO and serum levels of the total oxidative and anti-oxidative capacity (TOC and TAC) in cancer breast patients either newly diagnosed or metastatic cases in comparison to patients with benign breast tumours in order to add more knowledge to their possible role in these patients groups.

In our study, serum total anti-oxidant capacity (TAC) showed significant higher level in both cancer breast groups (N and M) as compared to the benign group (B) while, it showed no significant difference between the three patient groups (N, M and B) and the control group. However, total oxidative capacity TOC showed only significantly higher level in metastatic group than newly diagnosed group. 

Kanchan et al., (2016) study, observed significantly higher serum levels 8-OHdG as indicator of total oxidative capacity TOC in the all cancer breast patients in comparison with benign breast disease patients and the control group. A few studies have also reported an elevated level of this oxidative biomolecule in breast carcinogenesis and benign breast diseases, which might be involved in cancer breast initiation (Karihtala et al., 2011; Karki et al., 2015).

Opposite to our findings, they observed a substantial decrease in total antioxidant status and in non-enzymatic antioxidant profile in the malignant group, indicating increased use of serum antioxidants in response to an enhanced level of oxidative damage in these patients. Another study reported by Yuvaraj et al., (2008) showed that the different circulating enzymatic and non-enzymatic antioxidants were found to be low in a group of females with cancer breast.

This difference from our findings may due to difference in antioxidants measured and difference in patients’ characteristics. Owing to cooperative interaction, total activity may be greater than the sum of the individual antioxidants. In consistence with our results, other antioxidant studies in females with cancer breast found higher levels of antioxidant substances. Rajneesh et al., (2008) showed a significant elevation in both enzymatic and non-enzymatic antioxidants in serum samples of 40 cancer breast patients.

Nitric oxide levels showed statistically significant higher values in the three groups of patients than the control group in addition it was significantly greater in in the cancer groups (N and M) than the benign group (B). IL18 showed only significant higher levels in the metastatic patient group than the controls most probably due to the role of IL18 in cancer progression migration, invasion and metastasis .

In Günel et al., (2002) study, the serum IL-18 concentrations of all breast carcinoma patients were greater than the control subjects. As in our study, the serum IL-18 levels were significantly higher in metastatic patients compared with non-metastatic patients. These elevated levels may reflect the degree of defense mechanisms against tumor growth and metastasis in patients with breast carcinoma. 

Similarly, in the same Günel et al., (2002) study, the levels of serum nitrate and nitrite were increased significantly in breast carcinoma patients compared with control subjects with no difference in between metastatic and non-metastatic patients. Eissa et al., (2005) also showed IL18 levels were highly significant in the metastatic group than the control groups. Derin et al., (2007) found significant higher levels of IL18 in cancer breast patients than control. Parikh et al., (2017) suggested that IL-18 may be used as a guide for prediction and therapeutic target of newly diagnosed and metastatic cancer breast. 

Correlations between the different studied parameters showed positive correlation between TOC and IL18 and between TAC and NO in the benign group, however there where negative correlation between TOC and each of TAC and NO in the newly diagnosed group .This goes with Rashad et al., (2013) who found higher levels of NO and lower levels of TAC in cancer breast patients than control this could explain the antioxidants activity in protection of cancer breast ,while on studying the correlation among the laboratory parameters in the whole cancer group N+M there was positive correlation between TAC and NO.They suggested that, the elevated levels of NO levels may be due to host defense mechanism against tumor growth. In addition, NO and NO-derived reactive nitrogen species cause mutations in cancer-related genes or protein post-translation changes, thus increasing the danger of cancer.

*Conclusions and Recommendations*

• Nitric oxide is a powerful molecule that participates in cancer pathogenesis. Nitric oxide levels in breast cancer groups showed statistically significant higher values.

• It has been found that Interleukin (IL)-18 has been found to have a modifying role in the immune defense against tumor cells .In this study IL18 showed only significant higher levels in the metastatic patient group than the controls.

• Serum IL-18 and NO activity can be used as a marker for evaluating disease activity inpatients with breast carcinoma.

• Clinical significance of these parameters should be investigated in patients with breast carcinoma.

• The findings in this present study suggested increased serum total antioxidant capacity (TAC) as well as total oxidative capacity (TOC) in patients with cancer breast. However TOC in the metastatic group was only significantly higher than the newly diagnosed group. 

• Oxidative stress and antioxidant activity should be used as biomarkers for breast cancer. 

• Our findings in this study warrant further research into larger sample sizes and multiple serum samples with different time intervals. 
